# Comparison of different criteria for rheumatic heart disease screening: an empirical study in Sierra Leone

**DOI:** 10.1186/s12872-026-05758-0

**Published:** 2026-03-19

**Authors:** Belén Fernández-de-Toro, Lucía Cobarro, Jorge Pérez-Martín, Francisco Javier Díez

**Affiliations:** 1 Universidad Nacional de Educación a Distancia (UNED), Madrid, Spain; 2 Hospital Universitario 12 de Octubre, Madrid, Spain

**Keywords:** Rheumatic heart disease (RHD), Screening guidelines, Sierra Leone

## Abstract

**Background:**

Rheumatic heart disease (RHD) affects approximately 40 million people, primarily in low- and middle-income countries. Transthoracic echocardiography is the gold standard for early detection of RHD. Yet, no single echocardiographic finding or combination of findings unequivocally confirms RHD, especially in subclinical stages. As a result, multiple diagnostic guidelines have been proposed.

**Objectives:**

To characterise the echocardiographic RHD findings and criteria included in five of those guidelines; to assess how these diagnostic criteria differ when applied to a concrete population, namely a cohort of high-school students in Sierra Leone; and, as a secondary objective, to provide an initial estimate of the burden of RHD in this country.

**Methods:**

Our study has two parts. First, we performed a qualitative comparison of five major echocardiographic guidelines, mapping every diagnostic criterion into four roles (required, sufficient, combined, or not considered) for each guideline. Second, we conducted a school-based screening in Sierra Leone. A cohort of 604 asymptomatic female students (aged 10–22 years) underwent a two-phase echocardiographic protocol. We quantified regurgitant jet characteristics and morphological signs according to the criteria of the compared guidelines.

**Results:**

Guideline discrepancies were substantial, particularly in jet length thresholds and the diagnostic role of morphological features. These differences shifted individual classifications across categories. In our cohort, 13 students (2.2%) had findings beyond normal under at least one guideline, and positive cases ranged from 0.99% to 2.15% depending on the guideline. Using the World Heart Federation (WHF) 2023 criteria, 1.16% (95% CI: 0.47–2.37) were classified as positive.

**Conclusions:**

Variability in echocardiographic criteria influences RHD classification and prevalence estimation. Our empirical study shows that the use of rigid echocardiographic cut-offs may lead to discarding some cases suspicious of RHD, especially in the WHF 2023 screening guideline, because one unfulfilled criterion automatically leads to a negative diagnosis, regardless of the number and relevance of positive findings. We argue that a probabilistic causal model that ponders the numerical measurements and considers correlations among findings would be more appropriate for screening and diagnosis.

**Supplementary Information:**

The online version contains supplementary material available at 10.1186/s12872-026-05758-0.

## Background

Rheumatic heart disease (RHD) is caused by repeated episodes of acute rheumatic fever due to group A streptococcal infection. Around 40 million people currently live with RHD, and 300,000 die from the disease every year [1]. The standard of care is secondary prevention, consisting of screening programs for the detection of the disease when it is still asymptomatic, followed by antibiotic prophylaxis, usually with penicillin injections every 3 or 4 weeks [2].

Transthoracic echocardiography is the gold standard for early detection of RHD [3]. However, unlike most diagnostic tests used as gold standards for other diseases, echocardiography does not return a single finding or combination of findings that unequivocally confirms or discards the presence of RHD, but a broad set of observations (such as regurgitations and morphological anomalies, observed in several planes and with different techniques, such as 2D and Doppler) that cardiologists need to interpret and combine to determine whether there is a valvulopathy and, if so, whether it is of rheumatic origin. For this reason, several sets of criteria (guidelines) have been proposed to diagnose RHD.

Screening for early-stage RHD using echocardiography has consistently shown higher detection rates than auscultation alone [4]. Over the past two decades, several guidelines have been proposed to support screening and diagnosis, reflecting the need for standardised criteria that enable both the replication of findings and a meaningful comparison across studies. A second and equally important consideration is that the echocardiographic manifestations of subclinical RHD, the progression of disease and possible regional differences remain poorly defined. A clearer understanding of these aspects is crucial for refining diagnostic criteria and improving screening strategies worldwide.

The prevalence of RHD is higher in low-income countries (except for Australia and New Zealand, where it is endemic in some populations) [5], which do not have enough cardiologists to perform the screening. For this reason, in recent years, several studies have carried out the screening in two phases: first, briefly trained paramedical personnel examine the children using handheld probes; then, expert cardiologists examine the children with suspicious findings, either at the same school with a portable ultrasound machine or at a hospital with a standard sonograph [6–8].

Sierra Leone is a low-income West African country with a population of approximately 8.6 million and remains among the nations with the highest child and perinatal mortality rates [9]. According to estimates from the 2017 Global Burden of Disease study [10], the country has approximately 274,900 cases of prevalent cardiovascular disease (CVD) (3.7% of the population) and around 28,500 new cases every year. Among these, RHD and ischaemic heart disease are each estimated to account for about 23% of all CVD cases. However, the true burden and clinical impact of RHD in Sierra Leone remain unknown.

The objectives of this study are threefold. First, to conduct an in-depth analysis of the echocardiographic findings associated with RHD as defined across different guidelines. Second, to examine how different criteria diverge in practice when applied to a specific population, namely, a cohort of high-school students in Sierra Leone. Finally, a secondary aim was to obtain an initial understanding of the burden of RHD in this country by describing the proportion of students with findings consistent with RHD in a school-based setting.

## Methods

### Qualitative comparison of guideline criteria

We conducted a structured analysis of five diagnostic guidelines for the echocardiographic identification of RHD. We aimed to compare how each one defines pathological valvular findings and how these definitions influence the diagnostic labels assigned to each patient.

Among available frameworks, the 2012 World Heart Federation (WHF) criteria [11] remain the most widely used reference for the echocardiographic diagnosis of RHD [12, 13] and have served as the foundation for several subsequent guidelines. For this reason, we provide a more detailed description of these criteria.

This guideline defines morphological features of the mitral and aortic valves, together with detailed criteria for pathological mitral regurgitation (MR) and pathological aortic regurgitation (AR). It groups the echocardiographic findings in five categories:

1. MR-related criteria,
2. AR-related criteria,
3. mitral stenosis (MS),
4. mitral valve morphological features, and.
5. aortic valve morphological features.

These groups are not mutually exclusive, since morphological valve damage leads to haemodynamic consequences, such as regurgitation or stenosis, and the echocardiographic markers capture different manifestations of the same underlying pathological process.

To illustrate the structure of the WHF 2012 guideline, consider the criteria for *definite RHD* in individuals under 20 years. It can be diagnosed through one of four pathways:

A) pathological MR plus ≥ 2 morphological features of RHD of the mitral valve;
B) MS with a mean gradient ≥ 4 mmHg;
C) pathological AR plus ≥ 2 morphological features of the aortic valve; or.
D) *borderline RHD* of both the mitral and aortic valves.

The guideline later specifies detailed criteria for pathological regurgitation and morphological abnormalities. In contrast, it offers only general guidance for MS, without listing specific echocardiographic thresholds.

MS plays a central role in several RHD guidelines and is often considered a highly specific finding, but unlike regurgitation criteria—where explicit Doppler thresholds are provided—those RHD-specific frameworks offer limited formal definitions for MS beyond a mean gradient ≥ 4 mmHg. In clinical cardiology, MS diagnosis relies on a combination of morphological, haemodynamic and clinical features rather than on a single criterion [14, 15]. The lack of an explicit definition introduces ambiguity when interpreting MS within the different diagnostic frameworks.

The complexity of the WHF 2012 guideline limited its applicability for large-scale screening in low-resource settings [16], especially when the first phase of screening was done by paramedical personnel. This prompted the development of simplified frameworks. Thus, Beniwal et al. [17] proposed a quantitative score, Nunes et al. [16] developed a regression-based score for both screening and risk stratification based on only 5 findings, and Kotit [18] developed a simplified set of screening criteria. More recently, Rwebembera et al. published the 2023 WHF guidelines [19], based on a two-step approach that includes abbreviated criteria to identify suspicious cases (first phase) and confirmatory criteria for the definitive diagnosis of RHD (second phase). They also introduced a staging system to reflect severity and progression risk.

To systematically compare the five guidelines, we extracted every echocardiographic criterion they employ and mapped each finding into one of four diagnostic roles:

- required: the finding is indispensable for a positive classification; its absence automatically excludes a positive RHD diagnosis under that guideline;
- sufficient: the finding, if present, yields a positive RHD classification, without the need for additional criteria;
- weighted / combined: the finding contributes to the diagnostic decision but is neither necessary nor sufficient; it supports a positive classification only when considered alongside other findings.
- not considered: the finding is not mentioned in the guideline.

This structured framework allowed us to express heterogeneous diagnostic rules in a common language and compare them directly. The detailed criteria for the five guidelines are presented in Supplementary Material 1.

### Empirical comparison

#### Study design

Our protocol for RHD screening consists of two phases. The first one is a *basic examination* relying on five echocardiographic views: parasternal long axis, parasternal short axis at the levels of the mitral and aortic valves, apical four-chamber and apical five-chamber. This first examination was considered *positive* if any of the following criteria were met:

- MR observed in at least one view,
- AR observed in at least one view,
- thickening of the valve leaflets or chordae,
- restricted movement of the mitral valve leaflets, indicating reduced opening.

Positive cases immediately underwent a *detailed examination* to confirm and quantify the initial findings—measuring, for example, the length and duration of regurgitant jets—and to determine the presence of morphological signs. The list of possible findings for the detailed examination, shown in Supplementary Material 2, collects all the signs used in the guidelines mentioned above.

#### Study in Sierra Leone

A study was conducted in February 2024 at a female secondary school in Lunsar, Sierra Leone, which enrolled around 700 students. As it was uncertain whether it would be feasible to examine the entire student body, screening began with the older classes because the RHD prevalence increases with age and because these students were more likely to leave school soon.

A researcher from our team visited each classroom to explain the goals and procedures of the study and provided each student with an information sheet and an informed consent form. Each participant was assigned a randomised identification number that was used to record pseudonymised echocardiographic videos. The study protocol was approved by the Ethics Committee of the UNED on October 31st, 2023. A waiver of parental consent was granted by this committee because echocardiography is a non-invasive and innocuous procedure with no associated risk. It is generally accepted that children aged 10 years and above are competent to provide consent for interventions with high benefit and low risk [20]. In our study, those who wished to be examined came to the examination room where two female cardiologists were doing the echocardiograms. No one supervised which students went to the room and which did not, so participation was entirely voluntary.

The basic examination was done in parallel: one cardiologist used a Philips Lumify handheld probe connected to a Samsung Galaxy tablet and the other a Mindray MX7 portable ultrasound machine. In the case of suspicious findings, they jointly decided whether a detailed examination was necessary; if so, it was performed immediately using the Mindray MX7, which provides continuous-wave Doppler and superior image quality; both doctors jointly reviewed the echocardiographic findings.

## Results

### Qualitative comparison of guidelines criteria

The echocardiographic findings of all the guidelines are grouped according to the WHF 2012 guidelines into five broad categories: MR, AR, MS, mitral valve morphological signs and aortic valve morphological signs. The five guidelines classify patients into different levels: WHF 2012 and Beniwal et al. distinguish *normal*, *borderline* and *definite RHD*; Nunes et al. stratify individuals into *low-*, *intermediate-* and *high-risk* categories, depending on the numerical score; Kotit differentiates *possible* and *definite RHD*; and WHF 2023 adopts a four-staged system, from A to D.

#### Overall comparison

Table 1 provides an overview of how each guideline uses these five groups of findings to establish a diagnosis.

Three guidelines were explicitly designed for screening purposes: Nunes et al., Kotit and the screening criteria of WHF 2023. In the Nunes scoring system, no single group of findings is sufficient to move a case above the *low-risk* category (Table 1). All findings contribute only as weighted components, so that a *high-risk* classification requires the presence of multiple abnormalities.

Kotit applies a different logic. Mitral stenosis alone is sufficient for a *definite RHD* classification, although the guideline does not specify how MS should be confirmed, and only refers to “any degree of isolated stenosis”. In contrast, isolated AR is not sufficient even for *possible RHD* and must be accompanied by at least one morphological abnormality. Mild MR can be sufficient for *possible RHD*, although no explicit echocardiographic criteria are provided for this threshold. Isolated mitral valve thickening or isolated leaflet motion restriction are, however, sufficient for *possible RHD*.

In turn, the WHF 2023 screening criteria only require the presence of pathological MR, pathological AR or restricted motion *with* reduced opening of the mitral valve leaflets. Therefore, this guideline applies the lowest threshold for regurgitation-based findings, whereas Kotit applies the lowest threshold for mitral stenosis and mitral morphological abnormalities. In contrast, Nunes et al. consistently require a weighted combination of findings.

**Table 1:** Diagnostic role of major echocardiographic finding groups across the six guidelines

Finding group	WHF 2023 Screening	WHF 2023 Diagnosis	WHF 2012	Beniwal et al.	Nunes et al.	Kotit
Mitral regurgitation	sufficient for *positive*	sufficient for *stage A*	sufficient for *borderline RHD*	sufficient for *borderline RHD*	weighted	sufficient for *possible RHD*
Aortic regurgitation	sufficient for *positive*	sufficient for *stage A*	sufficient for *borderline RHD*	weighted	weighted	combined
Mitral stenosis	sufficient for *positive*	sufficient for *stage C*	sufficient for *definite RHD*	sufficient for *definite RHD*	weighted	sufficient for *definite RHD*
Mitral valve morphological signs	not considered	combined	sufficient for borderline RHD*	weighted	weighted	sufficient for *possible RHD*
Aortic valve morphological signs	not considered	combined	combined	weighted	weighted	sufficient for *possible RHD*

^*^Sufficient for *borderline RHD* classification only when at least two morphological abnormalities are present

#### Criteria for pathological mitral and aortic regurgitation

The detailed criteria used to define pathological MR are shown in Table 2. The diagnostic versions of the guidelines require stricter conditions than screening. For pathological MR, WHF 2023 diagnostic, WHF 2012 and Beniwal et al. require a jet length greater than 2 cm, pansystolic, visible in at least two views and with a velocity exceeding 3 m/s.

**Table 2:** Criteria used by the six guidelines to identify abnormal mitral regurgitation

Mitral regurgitation criterion	WHF 2023 Screening	WHF 2023 Diagnosis	WHF 2012	Beniwal et al.	Nunes et al.	Kotit
Jet length						
Any	-	-	-	-	-	sufficient for *possible RHD*
> 2 cm	required for pathological MR	required for pathological MR	required for pathological MR	required for pathological MR	weighted	-
Duration						
2 frames	required for pathological MR	-	-	-	-	-
Pansystolic	-	required for pathological MR	required for pathological MR	required for pathological MR	-	-
Observed in at least 2 views	-	required for pathological MR	required for pathological MR	required for pathological MR	-	-
Velocity >3 m/s	-	required for pathological MR	required for pathological MR	required for pathological MR	-	-

Among the screening-oriented frameworks, WHF 2023 screening applies the lowest threshold for MR, as a jet longer than 2 cm and visible in at least two consecutive frames is sufficient to classify a case as positive in the first phase. In Nunes et al., MR contributes to the score only if the jet length exceeds 2 cm, and no other regurgitation features are considered. In Kotit, mild MR is sufficient for *possible RHD*, although the criteria for mild severity are not specified.

A similar pattern is observed for AR (Supplementary Table S1). WHF 2023 screening requires only that AR be visible in at least two frames, making it the least restrictive framework for this finding. In contrast, Kotit requires AR to be accompanied by morphological abnormalities, and Nunes et al. treat it only as a weighted component that must be combined with additional findings.

#### Mitral stenosis and mitral valve morphological signs

Mitral stenosis emerges as one of the most specific findings across guidelines. As shown in Table 1, it is sufficient for *stage C* classification under WHF 2023 diagnostic and for *definite RHD* under WHF 2012, Beniwal et al. and Kotit. The score of Nunes et al. gives 3 points for anterior leaflet thickening of the mitral valve but does not explicitly mention mitral stenosis.

The criteria used to define mitral valve morphological abnormalities are summarised in Table 3. All the guidelines include thickening of the anterior mitral leaflet, chordal thickening, restricted anterior leaflet motion and restricted posterior leaflet motion. Nunes et al. only include thickening of the AML and excessive anterior leaflet motion. Excessive anterior leaflet motion is included in the WHF 2012 and 2023 guidelines and in Beniwal et al., but not in Kotit. Doming of the anterior leaflet is included by Beniwal and Kotit, while reverse doming is specific to Beniwal, and fusion of the subvalvular apparatus or commissures is specific to Kotit.

**Table 3:** Criteria used by the six guidelines to identify abnormal mitral morphological signs

Mitral morphological signs	WHF 2023 Screening	WHF 2023 Diagnosis	WHF 2012	Beniwal et al.	Nunes et al.	Kotit
Thickening						
AML	-	combined	combined	weighted	weighted	sufficient for *possible RHD*
Chordae	-	combined	combined	weighted	-	sufficient for *possible RHD*
Motion						
Restricted AML	-	combined	combined	weighted	-	sufficient for *possible RHD*
Restricted PML	-	combined	combined	weighted	-	sufficient for *possible RHD*
Excessive AML	-	combined	combined	weighted	weighted	-
Doming of AML	-	-	-	weighted	-	combined
Reverse doming of AML	-	-	-	weighted	-	-
Fusion of subvalvular apparatus or/and commissures	-	-	-	-	-	combined

Importantly, the diagnostic role assigned to these morphological signs differs substantially. For WHF 2012 and Kotit, certain mitral morphological abnormalities can be sufficient for diagnosing RHD even in the absence of regurgitation. In contrast, WHF 2023 diagnostic, Beniwal et al., and Nunes et al. require morphological signs in combination with regurgitation or other findings.

### Results of the study in Sierra Leone

In total, 604 students were examined. Their age ranged from 10 to 22 years, with a mean age of 15.0 years and a standard deviation of 2.0 years. All of them were asymptomatic. Twenty-three proceeded to the detailed examination. Thirteen (2.2% of the total population), ranging in age from 14 to 22 years, with a mean of 15.9, met the criteria to qualify as other than *normal* according to at least one guideline.

Table 4 shows the echocardiographic findings for these 13 subjects. All of them had at least mild MR, visible in two planes, with a jet length > 1 cm. Eight (1.3%) had a jet length > 2 cm, and 10 (1.7%) had a pansystolic mitral jet. Two students (0.3%) presented mild AR, with a regurgitant jet length > 1 cm; in one case, the regurgitation was visible in two planes but not pandiastolic, while in the other, it was pandiastolic but visible in only one plane. There were no cases of AR without concurrent MR.

**Table 4:** Findings in the 13 subjects who qualified as other than *normal* according to at least one guideline

	Mitral valve												Aortic valve				
Subject	Any MR?	Jet length>1 cm?	Jet length>2 cm?	2 frames?	Pansystolic?	2 views?	Thick AML	Thick PML	Thick chordae	Restricted motion	Excessive motion	Doming	Any AR?	Jet length>1 cm?	2 frames?	Pandiastolic?	2 views?
#1	Y	Y	N	Y	Y	Y	Y	N	N	N	N	N	N	N	N	N	N
#2	Y	Y	N	N	N	Y	Y	N	Y	N	Y	N	N	N	N	N	N
#3	Y	Y	N	Y	N	Y	Y	Y	Y	N	Y	N	N	N	N	N	N
#4	Y	Y	N	Y	Y	Y	Y	N	N	N	N	N	Y	Y	Y	N	Y
#5	Y	Y	N	Y	Y	Y	Y	N	N	N	N	N	Y	Y	Y	Y	N
#6	Y	Y	Y	Y	N	Y	Y	N	Y	N	N	N	N	N	N	N	N
#7	Y	Y	Y	Y	Y	Y	N	N	N	N	N	N	N	N	N	N	N
#8	Y	Y	Y	Y	Y	Y	N	N	N	N	N	N	N	N	N	N	N
#9	Y	Y	Y	Y	Y	Y	N	N	N	N	N	N	N	N	N	N	N
#10	Y	Y	Y	Y	Y	Y	N	N	Y	N	N	N	N	N	N	N	N
#11	Y	Y	Y	Y	Y	Y	Y	N	N	N	N	N	N	N	N	N	N
#12	Y	Y	Y	Y	Y	Y	Y	N	N	N	N	Y	N	N	N	N	N
#13	Y	Y	Y	Y	Y	Y	Y	N	Y	N	Y	N	N	N	N	N	N

Abbreviations: *Y* yes, *N* no

Ten subjects (1.7%) had mitral morphological findings, all of them having at least mild MR. Nine (1.5%) had thickening of the anterior mitral leaflet (AML), with one also showing thickened posterior leaflet (PML); the other case only had thick chordae. There were 5 cases (0.8%) of subvalvular thickening, 3 (0.5%) of excessive leaflet tip motion, and 1 case (0.2%) of doming. Five students (0.8%) had only one morphological sign, 2 (0.3%) presented two signs, and 3 (0.5%) had three. Only in 1 case (0.2%) was there a morphological sign (chordal thickening) without leaflet thickening. In all the other cases with at least one mitral morphological sign, leaflet thickening was present. Despite these findings, no patient had a mean peak gradient > 4.0 mmHg, the only quantitative criterion for mitral stenosis. No subject presented morphological signs in the aortic valve either.

Table 5 presents the classification of these 13 patients according to the five guidelines. The two WHF guidelines take into account the patient’s age, with a threshold at 10 years for WHF 2023 and 20 years for WHF 2012. Among the 13 patients, only #4, who is 22 years old, exceeds this threshold.

**Table 5:** Diagnosis of RHD according to different guidelines

Subject	WHF 2023		WHF 2012		Beniwal et al.		Nunes et al.		Kotit
	Scr.	Diag.							
#1	Neg.	Norm.	Norm.	-	Norm.	0.5	Low	3	Def.
#2	Neg.	Norm.	Bord.	A	Bord.	1.5	Low	6	Def.
#3	Neg.	Norm.	Bord.	A	Bord.	1.5	Low	6	Def.
#4	Pos.	Norm.	Norm.	-	Norm.	0.5	Inter.	8	Def.
#5	Pos.	Norm.	Norm.	-	Norm.	0.5	Inter.	8	Def.
#6	Pos.	Norm.	Bord.	A	Bord.	1	Inter.	9	Def.
#7	Pos.	A	Bord.	B	Bord.	1	Low	6	Poss.
#8	Pos.	A	Bord.	B	Bord.	1	Low	6	Poss.
#9	Pos.	A	Bord.	B	Bord.	1	Low	6	Poss.
#10	Pos.	B	Bord.	B	Bord.	1.5	Low	6	Poss.
#11	Pos.	B	Bord.	B	Bord.	1.5	Inter.	9	Def.
#12	Pos.	B	Bord.	B	Def.	2	Inter.	9	Def.
#13	Pos.	B	Def.	A	Def.	2.5	High	12	Def.

Abbreviations: *Neg* negative,  *Pos* positive,  *Norm* normal,  *A* stage A, *B* stage B, *Bord.* borderline, *Def.* definite,  *Inter* intermediate

Finally, it is worth noting that, even though the two versions of the WHF guidelines (2012 and 2023) use the same criteria for regurgitation and morphology, they combine them differently, which may result in discrepant diagnoses. Thus, patients #2, #3, and #6 in our study were considered *borderline* according to the 2012 guidelines but *normal* according to the new ones, due to the absence of pathological regurgitation. On the contrary, patients #10 to #12, classified as *stage B* by this version, were only *borderline* for the old one because they only had one morphological sign. For a case-by-case analysis, see Supplementary Material 3.

According to the WHF 2012 guidelines, the prevalence in our cohort is 0.17% (95% CI: 0.0%–0.92%) for *definite RHD* and 1.66% (95% CI: 0.8%–3.02%) if we consider both *borderline* and *definite* as positive, while it is 1.16% (95% CI: 0.47%–2.37%) for the WHF 2023 guidelines (including the four stages, A to D). We calculated these intervals using the Clopper-Pearson exact method, which is appropriate in this case because we are dealing with an extreme proportion. With other guidelines, prevalence ranges from 0.99% to 2.15%, with very wide confidence intervals due to the small number of positive cases.

## Discussion

### Limitations of current guidelines

Mitral and aortic regurgitation play a central role in all the guidelines, but the cut-offs used to label them as pathological differ (see Table 2 and Supplementary Material Table S1). As expected from the qualitative analysis, borderline findings, such as non-pandiastolic AR or AR visible only in one plane, affected the diagnoses in our clinical study. For example, patients #4 and #5 were classified as *positive* under WHF 2023 screening due to the presence of AR, but *normal* under diagnostic frameworks because the regurgitation did not meet the stricter criteria for pathological AR. Similarly, patient #6, with an MR jet of 2.2 cm that was not pansystolic, was classified as *borderline* under WHF 2012 and Beniwal et al., but *negative* under the WHF 2023 diagnostic.

Our qualitative comparison also highlighted important differences in how guidelines treat mitral valve morphology (see Table 3 and Supplementary Material Table S2): in some frameworks, certain morphological abnormalities suffice for a positive classification even in the absence of pathological regurgitation. The range of recognised morphological signs also varies across guidelines.

Empirically, 10 of the 604 screened students (1.7%) presented mitral morphological abnormalities, most commonly thickened AML, followed by subvalvular involvement, excessive leaflet motion and doming. None of these cases presented morphological abnormalities without at least mild MR, although in several cases the regurgitation did not meet the criteria for pathology. Patients #1 to #6 illustrate this pattern: morphological abnormalities were present alongside mitral regurgitation that fell below some of the pathological thresholds. Patients #2 and #3, who presented more than two mitral morphological signs with non-pathological mitral regurgitation, were classified as *borderline* under WHF 2012 and Beniwal et al., but as *normal* under WHF 2023 diagnostic and Nunes et al. Conversely, patients #10 to #12, with one or two morphological signs combined with pathological mitral regurgitation, were classified as *stage B* under WHF 2023 diagnostic and as *borderline* or *definite* under other frameworks.

These findings illustrate the limitations of rigid echocardiographic cut-offs. Small differences in jet length, such as 1.9 versus 2.0 cm, can shift a patient’s classification from positive to negative. In two-phase screening strategies, such as WHF 2023, this rigidity is especially problematic. Decision theory dictates that the threshold for a positive result in the first phase should be lower (i.e., more lenient) than in the confirmatory phase, given the asymmetry in costs [21], since a false positive in phase one only leads to further cardiological evaluation, whereas a false positive in phase two may result in the initiation of secondary prophylaxis, which is painful and consumes resources.

A related problem is that in rule-based guidelines, including both versions of WHF 2023, an unfulfilled criterion automatically leads to a negative diagnosis, regardless of the number and relevance of positive findings. In our cohort, patient #3, who had a mitral regurgitation jet of 1.7 cm and multiple morphological abnormalities, and patient #4, who had a 1.5 cm pansystolic jet and near-pathological aortic regurgitation, would have been discarded in the first phase under the strict > 2 cm screening threshold, despite other concerning features. These cases illustrate the potential for overly rigid criteria to miss individuals who may warrant closer follow-up.

Score-based guidelines avoid the problem of peremptory criteria, because a collection of positive findings may compensate for the absence of others, but they still suffer from the cut-offs problem. For example, Beniwal et al. give 2 points to a mitral diastolic gradient of 4 mmHg, which suffices to diagnose RHD, and Nunes et al. give 6 points to a MR jet of 2 cm, but slightly lower values receive no points. Furthermore, the score of Beniwal et al. does not represent true probabilities. Only the model of Nunes et al., based on logistic regression, outputs an explicit probability of disease and progression risk. However, it is limited to five echocardiographic signs. Adding other findings would increase the accuracy of the model, but to prevent overestimation of probabilities, correlations must be considered, which is not trivial in logistic regression. Another limitation of scoring systems is that they assign a single number to each finding, whereas two numbers should be assigned: sensitivity and specificity. In probabilistic diagnosis, the presence of a specific finding tends to confirm the presence of a disease, while the absence of a sensitive finding tends to rule it out. These two parameters cannot be reduced to a single weight because a sign can be highly sensitive yet not specific, highly specific yet not sensitive, etc.

These concerns align with those raised by Hunter et al. [22], who highlighted that, although the WHF 2012 criteria helped standardise RHD research, important methodological limitations remained, and the natural history of subclinical RHD is still incompletely understood. In a subsequent study [23], the same group proposed and tested a novel set of criteria in patients with proven acute rheumatic fever, emphasising the need to characterise the morphological features of mitral regurgitation better and to clarify its aetiology. This effort to better distinguish the origin of MR illustrates the importance of moving towards approaches that integrate multiple sources of information.

One way forward would be to build a causal probabilistic model based on expert knowledge and data. This could take the form of a Bayesian network or an influence diagram and would be able to incorporate many findings of different relevance while accounting for correlations among them.

### Impact on the diagnosis and prevalence estimation of RHD

Our empirical study confirms that using different criteria has a significant impact in practice. In our cohort of 604 students, prevalence ranges from 0.99% to 2.15%, depending on the guideline (with wide confidence intervals due to the small number of positive cases, which is a limitation of our study). Specifically, WHF 2012 yields a prevalence of 0.17% for *definite RHD*, rising to 1.66% when *borderline* cases are included, while the WHF 2023 guidelines, which constitute the consensus of experts, result in a prevalence of 1.16%. These discrepancies underscore that prevalence estimates derived from different guidelines may not be interchangeable. This observation is particularly relevant in light of recent meta-analyses, where pooled prevalence estimates often combine studies that use different diagnostic criteria, potentially explaining the high levels of heterogeneity reported [24, 25].

Even more importantly, the choice of guideline may affect the diagnosis of individual subjects, and consequently, the treatment they receive. Since both false negatives and false positives incur significant costs, the substantial discrepancies shown in Table 5 have a significant impact on clinical practice. One might argue that the 2023 WHF guidelines, which are based on the most recent evidence and the consensus of a large group of experts, take precedence over previous recommendations. However, it is worth noting that some of the subjects in our study classified as *normal* by WHF 2023 had a Nunes score of 8 or 9 (*intermediate risk*), while WHF 2023 classified as *A* or *B* some subjects with a score of 6 (*low risk*). Given that the score of Nunes et al. was derived from a study involving 9,501 children and validated on two cohorts totalling 7,539 children, we question whether the recent WHF guideline is necessarily more accurate than the score.

### Can this estimation of prevalence be extrapolated to Sierra Leone?

Although our study was not designed to estimate the prevalence of RHD in Sierra Leone, in the absence of any other studies on this topic, it is worth discussing whether our results are representative of this country. Firstly, the prevalence derived from our study may be overestimated due to gender bias, as most studies have found that this disease affects females more than males [26]. Additionally, prevalence increases with age, so our study likely yielded a higher estimate than if it had enrolled children aged 5 to 16 or 18 years [27]. A possible bias in the opposite direction is that our screening took place in a private school, where prevalence is likely lower than in other schools [28] and almost certainly lower than among out-of-school teenagers in the same town. Another key limitation, common to most studies about RHD, is that we have not followed up suspicious cases, which, together with the absence of a gold standard, makes it impossible to determine which patients really have the disease.

In any case, our results align with reports from other sub-Saharan countries. A systematic review and meta-analysis of 21 studies conducted under WHF 2012 criteria reported an estimated 25.5 cases per 1,000 population (≈ 2.55%) for latent RHD in Africa, with school-based screening yielding approximately 7.9 cases per 1,000 (≈ 0.79%) [5].

## Conclusions

Current echocardiographic guidelines, usually based on expert judgment, differ significantly in the cut-offs for echocardiographic measurements and the weighting of morphological features. Our study in Sierra Leone shows that discrepancies are not just conceptual: they can also affect the estimation of prevalence and, more importantly, the diagnosis and treatment of patients, as some individuals can receive unnecessary treatments while others are left at risk of disease progression. Future work should explore probabilistic models, which are more flexible and can integrate multiple echocardiographic signs, consider sensitivity and specificity, and reflect the uncertainty inherent in subclinical disease. Such approaches could improve diagnostic accuracy, guide follow-up strategies and support better decision-making in resource-limited settings.

## Supplementary Information

Supplementary Material 1. Summary of the guidelines.

Supplementary Material 2. Finding sheet for our study.

Supplementary Material 3. Case-by-case analysis.

Supplementary Material 4. Table S1: Criteria used by the six guidelines to identify abnormal aortic regurgitation. Table S2: Criteria used by the six guidelines to identify abnormal aortic morphological signs.

## Data Availability

The datasets used and analysed during the current study are available from the corresponding author on reasonable request.

## Abbreviations

AML: Anterior Mitral Leaflet
AR: Aortic Regurgitation
AS: Aortic Stenosis
AV: Aortic Valve
CVD: Cardiovascular Disease
MR: Mitral Regurgitation
MS: Mitral Stenosis
MV: Mitral Valve
PML: Posterior Mitral Leaflet
RHD: Rheumatic Heart Disease
WHF: World Heart Federation
