# Supplementary material for: Comparison of different criteria for rheumatic heart disease screening: an empirical study in Sierra Leone
Source: BMC Cardiovasc Disord. 2026 Mar 19;26:364. doi: 10.1186/s12872-026-05758-0 (PMC13122888; doi:10.1186/s12872-026-05758-0)
Supplement: Supplementary file 1 — Supplementary Material 1. Summary of the guidelines. [file 12872_2026_5758_MOESM1_ESM.docx]

**Supplementary Material 1. Summary of the guidelines**

**WHF 2012** (11)

Criteria for pathological regurgitation:

*Pathological MR (all four Doppler echocardiographic criteria must be met)*

1. Seen in two views
2. In at least one view, jet length ≥ 2 cm
3. Velocity ≥ 3 m/s for one complete envelope
4. Pan-systolic jet in at least one envelope

*Pathological AR (all four Doppler echocardiographic criteria must be met)*

1. Visible / Observable in two views
2. In at least one view, jet length ≥ 1 cm
3. Velocity ≥ 3 m/s in early diastole
4. Pan-diastolic jet in at least one envelope

*Morphological features of the MV*

- AMVL thickening:
  - ≥3 mm for individuals aged ≤20 years,
  - ≥4 mm for individuals aged 21–40 years.
  - ≥5 mm for individuals aged >40 years.
- Chordal thickening
- Restricted leaflet motion
- Excessive leaflet tip motion during systole

*Morphological features of the AV*

- Irregular or focal thickening
- Coaptation defect
- Restricted leaflet motion
- Prolapse

Echocardiographic criteria for individuals aged ≤20 years:

*Normal Echocardiographic Findings (all of A, B, C, and D)*

1. MR that does not meet all four Doppler echocardiographic criteria (physiological MR)
2. AR that does not meet all four Doppler echocardiographic criteria (physiological AR)
3. One morphological feature of RHD of the MV without any associated pathological stenosis or regurgitation
4. One morphological feature of RHD of the AV without any associated pathological stenosis or regurgitation.

*Borderline RHD (either A, B, or C)*

1. At least two morphological features of the MV without pathological MR or MS
2. Pathological MR
3. Pathological AR.

*Definite RHD (either A, B, C, or D)*

1. Pathological MR and at least two morphological features of the MV
2. MS mean gradient ≥ 4 mmHg
3. Pathological AR and at least two morphological features of the AV
4. Borderline disease of both the AV and MV.

Echocardiographic criteria for individuals aged >20 years:

*Definite RHD (either A, B, C, or D)*

1. Pathological MR and at least two morphological features of RHD of the MV
2. MS mean gradient ≥ 4 mmHg
3. Pathological AR and at least two morphological features of RHD of the AV, only in individuals aged <35 years
4. Pathological AR and at least two morphological features of RHD of the MV

**Beniwal et al. 2015** (17)

*Functional deficits*

- Mitral stenosis with mean gradient during diastole ≥ 4 mmHg: 2 points
- Pathological mitral regurgitation (according to WHF 2012 criteria): 1 point
- Pathological aortic regurgitation (according to WHF 2012 criteria): 0.5 points

*Morphological abnormalities*

- Thickening of AML and/or PML: 0.5 points
- Thickened chordae: 0.5 points
- Restricted movement of AML and/or PML: 0.5 points
- Excessive leaflet tip movement of AML and/or PML: 0.5 points
- Doming of AML during diastole and/or reverse doming (prolapse) in systole: 0.5/0.5 points
- Aortic valve leaflet thickening: 0.25 points
- Prolapse of aortic leaflets: 0.25 points
- Restricted movement of aortic leaflets: 0.25 points

Cases with a cumulative score ≥1 and <2 are classified as *borderline RHD* and those with a score ≥ 2 as *definite RHD*.

**Nunes et al. 2019** (16)

*Functional deficits*

- Regurgitation jet length ≥ 2 cm for mitral valve: 6 points
- Any regurgitation for aortic valve: 5 points

*Morphological abnormalities*

- Anterior leaflet thickening (MV): 3 points
- Excessive leaflet tip motion (MV): 3 points
- Irregular or focal thickening of aortic valve: 4 points

The final scoring system results in a total score of 0 to 21 points. The risk categories are defined as follows:

- *Low-risk:* 0 to 6 points
- *Intermediate-risk:* 7 to 9 points
- *High-risk:* ≥10 points

**Kotit 2021** (18)

*Definite RHD* is diagnosed when there is any degree of isolated stenosis, or any degree of regurgitation in combination with at least one of these morphological findings,

- valvular thickening
- doming of valve cusps
- fusion of subvalvular apparatus or/and commissures
- leaflet shortening and restriction,

*Possible RHD* is diagnosed in the presence of any of these findings:

- isolated valvular thickening
- isolated leaflet restriction
- isolated mitral regurgitation, even mild.

**WHF 2023** (19)

This guideline considers two phases for RHD screening. The first one can be done by paramedical personnel with handheld probes. It is based on the detection of three abnormalities (for individuals aged ≤20 years):

- Pathological MR, defined as the presence of a regurgitant jet observed in at least two consecutive frames in at least one view with a minimum length of 1.5 cm for patients weighing <30 kg and 2.0 cm for patients weighing ≥30 kg
- Pathological AR, defined as the presence of a regurgitant jet (of any length) observed in at least two consecutive frames in at least one view
- Mitral stenosis, detected by restricted leaflet motion with reduced valve opening.

The result is *positive* when any of the three is present.

The examination in the second phase must be performed by a cardiologist or an expert sonographer using a portable or a standard ultrasound machine, and requires additional conditions, based on continuous-wave Doppler measurements, to confirm each of the three abnormalities:

- Pathological MR: the jet must be pansystolic and have velocity ≥3.0 m/s for one complete envelope.
- Pathological AR: the jet must be pandiastolic and have a velocity ≥3.0 m/s in early diastole.
- Mitral stenosis: the mean peak gradient must be ≥4.0 mmHg.

Therefore, some abnormalities found in the first phase can be ruled out in the second.

The morphological findings for this phase are:

Mitral valve:

- Valve apparatus thickening (AML, chordal thickening).
- Valve mobility abnormalities (restricted AML/PML motion in diastole, excessive AML tip motion in systole).

Aortic valve:

- Cusp thickening.
- Cusp prolapse.
- Restricted cusp motion.
- Coaptation defect in diastole.

Staging of RHD detected by echo echocardiography.

- **Stage A**: Mild pathological MR or AR without morphological features.
- **Stage B**: Either both MR and AR, or any pathological regurgitation plus ≥1 (≤20 years) or ≥2 morphological features (>20 years).
- **Stage C**: Moderate/severe MR, moderate/severe AR, any MS or AS, pulmonary hypertension, decreased LV systolic function.
- **Stage D**: Moderate/severe MR, moderate/severe AR, any MS or AS, pulmonary hypertension, decreased LV systolic function.
