# Supplementary material for: Comparison of different criteria for rheumatic heart disease screening: an empirical study in Sierra Leone
Source: BMC Cardiovasc Disord. 2026 Mar 19;26:364. doi: 10.1186/s12872-026-05758-0 (PMC13122888; doi:10.1186/s12872-026-05758-0)
Supplement: Supplementary file 2 — Supplementary Material 2. Finding sheet for our study. [file 12872_2026_5758_MOESM2_ESM.docx]

**Supplementary Material 2. Finding sheet for our study**

**Basic examination**

In our study, the basic examination was done by two cardiologists, using a handheld probe, or a portable ultrasound machine, or both. In other studies, it could be done by nurses or technicians.

- PLAX
  - Mitral regurgitation: no / trivial / mild / moderate / severe
    - In more than one frame: no / yes
    - Eccentric: no / yes
    - Jet length (mm):
  - Restricted MV leaflet motion and reduced opening: no / dubious / yes
  - Aortic regurgitation: no / trivial / mild/ moderate / severe
    - In more than one frame: no / yes
    - Eccentric: no /yes
    - Jet length (mm):
- PSAX-MV
  - Mitral regurgitation: no / yes
- PSAX-AV
  - Morphology: normal / bicuspid
  - Aortic regurgitation: no / yes
- A4C
  - Mitral regurgitation: no / trivial / mild / moderate / severe
    - In more than one frame: no / yes
    - Eccentric: no / yes
    - Pan-systolic: no / yes
    - Jet length (mm):
  - Restricted MV leaflet motion and reduced opening: no / dubious / yes
- A5C
  - Aortic regurgitation: no / trivial / mild / moderate / severe
    - In more than one frame: no / yes
    - Eccentric: no / yes
    - Pan-diastolic: no / yes
    - Jet length (mm):

**Detailed examination**

In our study, the detailed examination of subject with suspicious findings in the basic examination was also done by the two cardiologists, using the portable ultrasound machine.

- If there is MR:
  - Pan-systolic jet in at least one envelope: no / yes
  - MR velocity in PLAX (m/s):
  - MR velocity in A4C (m/s):
- If there is AR:
  - Pan-diastolic jet in at least one envelope: no / yes
  - AR velocity in early diastole in PLAX (m/s):
  - AR velocity in early diastole in in A5C (m/s):
- Mitral valve morphology
  - Thickened leaflets: none / AML / PML / both
  - Thickened chordae: no / yes
  - Restricted movement of AML and/or PML: no / yes
  - Commissural fusion: no / yes
  - Excessive leaflet tip movement of AML and/or PML: no / yes
  - Doming of AML during diastole: no / yes
  - Reverse doming (prolapse) in systole: no / yes
- Aortic valve morphology
  - Leaflet thickening: no / yes
  - Prolapse of aortic leaflets: no / yes
  - Restricted movement of leaflets: no / yes
- If there is suspicion of mitral stenosis:
  - Mean gradient during diastole (mmHg):

Mitral valve area in 2D (cm^2^):
