# Supplementary material for: Comparison of different criteria for rheumatic heart disease screening: an empirical study in Sierra Leone
Source: BMC Cardiovasc Disord. 2026 Mar 19;26:364. doi: 10.1186/s12872-026-05758-0 (PMC13122888; doi:10.1186/s12872-026-05758-0)
Supplement: Supplementary file 3 — Supplementary Material 3. Case-by-case analysis. [file 12872_2026_5758_MOESM3_ESM.docx]

**Supplementary Material 3. Case-by-case analysis**

**Patient #1** has a 1.5 cm MR jet, which is pansystolic and visible in more than one plane, and one mitral morphological sign, thickened AML. Kotit’s guideline considers this patient *positive* for RHD because there is regurgitation and one morphological sign. All the other guidelines consider this case *negative* because the MR jet length is less than 2 cm and/or there are less than two morphological signs.

**Patients #2 and #3** present non-pansystolic MR jets between 1 and 2 cm, visible in at least two planes, no AR and more than two morphological signs on the mitral. WHF 2012 and Beniwal et al. classify them as *borderline* because they display two or more morphological signs but do not meet their criteria for pathological MR. The WHF 2023 and Nunes et al. classify them as *normal*, despite the presence of 3 or 4 morphological signs, because the regurgitations do not meet the criteria for being pathological.

**Patients #4 and #5** also have MR jets between 1 and 2 cm visible in at least two planes, but these are pansystolic. They also exhibit AR, not pandiastolic in case #4, and visible in just one plane in #5. Both patients have AML thickening. They meet the WHF 2023 screening criteria due to the presence of AR, but not the diagnostic criteria for pathological MR or AR, so they are finally classified as *normal*. According to WHF 2012 and Beniwal et al., these regurgitations do not qualify as pathological and, having only one morphological sign, these patients are not even considered *borderline*. In contrast, Nunes et al., who give 5 points for the presence of any AR, regardless of jet length or duration, and 3 points for the presence of AML thickening, assign them an *intermediate risk*. These patients are *definite* for Kotit owing to having MR, regardless of the jet length, and one morphological sign.

**Patient #6** has a non-pansystolic MR jet measuring 2.2 cm, observable in 2 views, along with two morphological signs on the mitral valve. This patient is therefore *borderline* for Beniwal et al. (who assign 1 point for MR) and for WHF 2012, and *negative* for WHF 2023 because the jet is not pansystolic. Nunes et al. assign this patient an *intermediate risk* (9 points).

**Patients #7, #8, and #9** exhibit MR jets exceeding 2 cm, which are pansystolic and visible in two planes. As a result, they are all classified as *stage A* for WHF 2023 and *borderline* for WHF 2012 and for Beniwal et al. However, due to the absence of morphological signs on the regurgitant valve, Nunes et al. assign them a *low risk*, and Kotit considers them as *possible RHD*.

**Patients #10 and #11** show MR jets over 2 cm, pansystolic and visible in two planes, as well as one morphological sign on the mitral valve. Consequently, they are classified as *stage B* by WHF 2023, and *borderline* by WHF 2012 and by Beniwal et al.

Nunes et al. assign #10 a *low risk* (6 points) and Kotit classifies the patient as *possible RHD*—as occurred with patients #7, #8, and #9, who lacked morphological signs on the mitral valve—because these guidelines do not consider chordal thickening a morphological sign of RHD. Conversely, both guidelines recognize AML thickening as a sign, which leads Nunes et al. to assign #11 an *intermediate risk* (9 points) and Kotit to classify it as *definite RHD*.

**Patient #12** presents with a MR jet measuring 2.3 cm, pansystolic and visible in two planes, and two morphological signs on the mitral valve: thickened AML and doming. All the authors classify this case as *positive*.

**Patient #13** exhibits a MR jet of 3.0 cm, pansystolic and visible in two planes, as well as three morphological signs on the mitral valve: thickened AML, thickened chordae and excessive leaflet motion. All guidelines classify this case as *positive*. Nunes et al. assign it a *high risk*, with 12 points.
