# Supplementary material for: Comparison of different criteria for rheumatic heart disease screening: an empirical study in Sierra Leone
Source: BMC Cardiovasc Disord. 2026 Mar 19;26:364. doi: 10.1186/s12872-026-05758-0 (PMC13122888; doi:10.1186/s12872-026-05758-0)
Supplement: Supplementary file 4 — Supplementary Material 4. Table S1: Criteria used by the six guidelines to identify abnormal aortic regurgitation. Table S2: Criteria used by the six guidelines to identify abnormal aortic morphological signs. [file 12872_2026_5758_MOESM4_ESM.docx]

**Supplementary Material Table S1. Criteria used by the six guidelines to identify abnormal aortic regurgitation.**

| **Aortic regurgitation criterion** | **WHF 2023**  **Screening** | **WHF 2023**  **Diagnosis** | **WHF 2012** | **Beniwal *et al.*** | **Nunes *et al.*** | **Kotit** |
| --- | --- | --- | --- | --- | --- | --- |
| Jet length |  |  |  |  |  |  |
| Any | required  suspicious AR | - | - | - | weighted | required  suspicious AR |
| >1cm | - | required  suspicious AR | required  suspicious AR | required  suspicious AR | - | - |
| Duration |  |  |  |  |  |  |
| 2 frames | required  suspicious AR | - | - | - | - | - |
| Pandiastolic | - | required  suspicious AR | required  suspicious AR | required  suspicious AR | - | - |
| Observed in at least 2 views |  | required  suspicious AR |  |  |  |  |
| Velocity >3m/s | - | required  suspicious AR | required  suspicious AR | required  suspicious AR | - | - |

**Supplementary Material Table S2. Criteria used by the six guidelines to identify abnormal aortic morphological signs.**

| **Aortic morphological signs** | **WHF 2023**  **Screening** | **WHF 2023**  **Diagnosis** | **WHF 2012** | **Beniwal *et al.*** | **Nunes *et al.*** | **Kotit** |
| --- | --- | --- | --- | --- | --- | --- |
| Subvalvular thickening | - | combined | combined | - | - | sufficient *possible RHD* |
| Leaflet motion restriction | - | combined | combined | weighted | - | sufficient *possible RHD* |
| Coaptation defect | - | combined | combined | - | - | - |
| Prolapse | - | - | combined | weighted | - | - |
